# Genome-Wide Association Analysis of Semen Characteristics in Piétrain Boars

**DOI:** 10.3390/genes15030382

**Published:** 2024-03-20

**Authors:** Henry Reyer, Ibrahim Abou-Soliman, Martin Schulze, Hubert Henne, Norbert Reinsch, Jennifer Schoen, Klaus Wimmers

**Affiliations:** 1 Research Institute for Farm Animal Biology (FBN), 18196 Dummerstorf, Germany; shawkidrc@yahoo.com (I.A.-S.); reinsch@fbn-dummerstorf.de (N.R.); wimmers@fbn-dummerstorf.de (K.W.); 2 Department of Animal and Poultry Breeding, Desert Research Center, Cairo 11753, Egypt; 3 Institute for Reproduction of Farm Animals Schönow, 16321 Bernau, Germany; m.schulze@ifn-schoenow.de; 4 BHZP GmbH, 21368 Dahlenburg-Ellringen, Germany; 5 Leibniz Institute for Zoo and Wildlife Research (IZW), 10315 Berlin, Germany; schoen@izw-berlin.de; 6 Institute of Biotechnology, Technische Universität Berlin, 10623 Berlin, Germany; 7 Faculty of Agricultural and Environmental Sciences, University of Rostock, 18059 Rostock, Germany

**Keywords:** Piétrain pig, sperm morphology, sperm motility, spermatogenesis, GWAS

## Abstract

Since artificial insemination is common practice in pig breeding, the quality and persistence of the semen are decisive for the usability of individual boars. In the current study, genome-wide association analyses were performed to investigate the genetic variability underlying phenotypic variations in semen characteristics. These traits comprise sperm morphology and sperm motility under different temporal and thermal storage conditions, in addition to standard semen quality parameters. Two consecutive samples of the fourth and fifth ejaculates from the same boar were comprehensively analyzed in a genotyped Piétrain boar population. A total of 13 genomic regions on different chromosomes were identified that contain single-nucleotide polymorphisms significantly associated with these traits. Subsequent analysis of the genomic regions revealed candidate genes described to be involved in spermatogenesis, such as *FOXL3*, *GPER1*, *PDGFA*, *PRKAR1B*, *SNRK*, *SUN1*, and *TSPO*, and sperm motility, including *ARRDC4*, *CEP78*, *DNAAF5*, and *GPER1*. Some of these genes were also associated with male fertility or infertility in mammals (e.g., *CEP78*, *GPER1*). The analyses based on these laboriously determined and valuable phenotypes contribute to a better understanding of the genetic background of male fertility traits in pigs and could prospectively contribute to the improvement of sperm quality through breeding approaches.

## 1. Introduction

The Piétrain breed is widely used as a terminal sire line in commercial breeding programs to obtain pigs with a good feed conversion efficiency, high muscularity, and leanness [1]. Besides growth and carcass traits, the reproductive performance of Piétrain is of particular importance to the selection index, although it is mainly represented by the number of live born piglets per boar [2,3]. Boar fertility traits and semen quality are valuable indicators for reproduction, as they influence sperm’s ability to reach and fertilize an oocyte [4]. These traits play a key role in the implementation of artificial insemination (AI), which is common practice in pig breeding [5]. The recording and application of a wide range of sperm and fertility parameters are conceivable. These include pure boar parameters such as ejaculate volume, sperm concentration, and total sperm number per ejaculate, which can be measured routinely [6]. More informative for the assessment of reproductive performance are the morphological characteristics of the sperm and so-called ‘stress tests’ [6]. The assessment of sperm motility as part of a stress test helps to evaluate the fertilization capacity and the resilience of the sperm to environmental changes. Indeed, the motility under stress conditions varies between normal and abnormal sperm groups and in the process of sperm senescence [7]. For boar semen, these data can assist in predicting the fertilization potential of ejaculates by evaluating sperm’s persistence to thermal stress, usually using computer-assisted sperm analysis (CASA) systems. Sperm motility testing following prolonged exposure to 38 °C simulates the duration for which sperm remain in the female genital tract. This allows conclusions to be drawn about their ability to fertilize an oocyte and their functional metabolism. A stress test at low temperatures, i.e., 6 °C, simulates a beneficial semen storage regimen that limits bacterial growth but could also affect the semen quality. Sperm motility testing allows the detection of even small differences between high-quality ejaculates. Furthermore, the semen quality index compiled from these measurements accurately reflects the requirements of AI and facilitates the prediction of fertility [8]. The disadvantage is that these parameters are complex to collect and are therefore not yet available for large boar populations.

Sperm quality is affected by many factors, above all the age of the boar and the respective pig breed [8]. The latter indicates that there is a considerable genetic contribution to sperm quality traits. In dairy and beef cattle, genetic parameters and heritability estimates have been determined for semen quality traits, suggesting that these traits can be improved by selective breeding [9]. In pigs, analysis of the economically most important breeds, including the Piétrain-based, Duroc-based, Large White-based and Landrace-based lines, showed the moderate heritability of sperm motility, sperm counts, and total morphological abnormalities [10]. A number of recent genetic studies in Duroc pigs identified several genomic regions and candidate genes that could contribute to the variability in semen quality traits [11,12,13,14]. Some of the QTL regions identified contained candidate genes such as *STRA8*, *UBB*, *DNM1*, and *CATSPER1*, which may be associated with spermatogenesis and fertilizing capacity. However, it was discussed that the QTL regions appear to be breed-specific, reflecting the complex genetic architecture of quantitative traits. This was reflected in a GWAS with sperm motility traits that included both Large White and Landrace pigs and revealed a number of QTL regions, while none of the regions overlapped between the two lines [15]. The mentioned studies provide insights into the genetic architecture of semen quality, while more complex phenotypes, e.g., from sperm motility tests under stress conditions, have not yet been applied in genome-wide analyses.

The aim of the present study was to identify the genomic regions associated with the boar parameters and phenotypes derived from complex sperm motility tests in a population of young Piétrain boars. Specifically, the sperm quality parameters with a proven impact on boar fertility [16,17] were assessed using two consecutive ejaculates per boar subjected to standard storage and cold and heat stress tests.

## 2. Materials and Methods

### 2.1. Ethics Statement

All the procedures involving animals were carried out in accordance with the guidelines and regulations according to the European Commission Directive for Pig Welfare and were approved by the animal welfare committee of the Institute for Reproduction of Farm Animals Schönow (IFN-2019-V-05). The semen sampling was carried out at BHZP Bösewig AI center (BHZP GmbH, Bösewig 21, 06905 Bad Schmiedeberg, Germany) during routine animal selection and semen production for AI purposes.

### 2.2. Animals and Phenotypes

The present study exploits data from a previous report evaluating the classical semen parameters and complex semen stress tests (heat resistance and cold resistance) in relation to fertility records after AI. The semen collection, semen processing, evaluation of sperm motility and sperm morphology, and flow cytometric assessment of the spermatozoa are described in detail in a previous publication [17]. Briefly, the fourth and fifth ejaculates of each Piétrain boar (n = 129) at an age between 8 and 9 months were collected and assessed for their ejaculate volume, sperm concentration, and fresh sperm motility on site in the boar stud before dilution and packaging in a standard Beltsville Thawing Solution (BTS) semen extender. The reference laboratory at IFN Schönow subsequently analyzed the sperm numbers per AI dose, sperm morphology, motility, mitochondrial activity, and acrosomal status (Table 1). The proportion of morphologically normal sperm and the droplet rate were determined according to phase-contrast microscopic examination of 200 spermatozoa (Jenaval, Carl Zeiss Jena, Jena, Germany). The mitochondrial activity and acrosome status were analyzed following double staining with propidium iodide and rhodamine 123 using a CytoFLEX S flow cytometer (Beckmann Coulter, Krefeld, Germany) with a 488 nm solid-state laser. The motility of the sperm was assessed utilizing the AndroVision (version 1.1, Minitube, Tiefenbach, Germany) CASA system coupled with a phase-contrast microscope (Axio Scope A1, Carl Zeiss Microscopy, Germany). The spermatozoa were defined as motile based on the default setting of the manufacturer for boar semen. The sperm motility of the liquid-preserved semen was examined repeatedly on days 1, 2, and 3 after standard storage (17 °C). In addition, their motility was analyzed following cold (after 6 °C storage for three days) and heat resistance tests (38 °C incubation for 30 and 300 min after seven days storage at 17 °C) [17]. All the samples were collected and analyzed between January and December 2019. To account for environmental variations in the pig station, each sample pair was assigned a sampling season covering the cold season from October to the end of March and the warm season from April to September.

The distribution of the data on the two ejaculates was checked using the Shapiro–Wilk normality test implemented in the stats R package, and comparison of the two ejaculates was performed accordingly using Student’s *t*-test or the Kruskal–Wallis rank sum test. A rank-based inverse normal transformation of the phenotypes was applied to mitigate any deviations from a normal distribution prior to the genome-wide association study (GWAS). In order to categorize adequate and reduced sperm motility after storage at 17 °C and after the heat resistance test (HRT), these two traits were dichotomized based on the distribution of the data measured for the 258 ejaculates (Figure S1). Corresponding cut points were set at 75% and 40%, respectively. Pearson’s correlation coefficients between traits were calculated using SAS version 9.4 (SAS Institute, Cary, NC, USA).

### 2.3. Genotypes and Genome-Wide Association Study

The genotype information for each boar was derived from DNA obtained from routine blood samples using a Porcine SNP60k BeadChip (Illumina, San Diego, CA, USA). The referenced SNP data were provided by BHZP Germany. SNPs with a minor allele frequency (MAF) < 0.05 and those not assigned a clear chromosomal position on the pig reference genome (Sscrofa11.1, version 108) or localized to sex chromosomes were removed, leaving 40,820 SNPs for further analyses. The genome-wide association analyses were performed after rank-based inverse normal transformation of the phenotypes using the RepeatABEL package in the R environment (https://cran.r-project.org/src/contrib/Archive/RepeatABEL, accessed on 22 November 2022) [18]. RepeatABEL was specifically developed for genetic analysis of repeated observations; thus, the transformed phenotypes of the 4th and 5th ejaculates were directly used as input for the GWAS. Analyses were conducted using a linear mixed model with SNP as a fixed effect, season as a fixed effect, and a random polygenic effect to consider the relatedness of individuals. For the polygenic effect, a genomic relationship matrix was calculated [19]. Repeated observations were considered for measurements of the sperm quality parameters from the consecutively sampled ejaculates (Table 1). In addition, for the total sperm motility after storage at 17 °C, the measurements on days 1, 2 and 3 were considered repeated observations, and for the heat resistance test assessed on day 7 of semen storage, the measurements taken after 30 min and 300 min at 38 °C were considered repeated observations.

The SimpleM script was used to estimate the number of independent statistical tests by performing a principal component analysis based on a composite linkage disequilibrium matrix from the SNP genotypes [20]. According to the 16,843 independent tests, the significance cut-offs were set at −log10(*P*) = 4.23 (1/16,843) for suggestive evidence and −log10(*P*) = 5.53 (0.05/16,843) for genome-wide evidence according to [21]. In this context, it should be noted that SNPs reported with suggestive evidence of an association have an increased risk of false positive findings. The GWAS results were visualized in Manhattan and QQ plots for each trait using R.

### 2.4. Detection of Candidate Genes

The detection of candidate genes was performed by identifying genes in the Ensembl database within 1 Mb windows around the significant SNPs. Genes that directly contain one of the trait-associated SNPs were taken as positional candidate genes. Genes with described functional relationships to the phenotypes were considered functional candidates, taking into account the available gene annotations from GeneCards (http://www.genecards.org, accessed on 4 September 2023) [22] and UniProt (http://www.uniprot.org, accessed on 4 September 2023).

## 3. Results

### 3.1. Phenotypic Data of Sperm Quality Traits

The studied semen quality traits comprised the boar parameters, sperm motility after different storage and stress conditions, and sperm morphology characteristics (Table 2). Comparison of the average values from the 4th and 5th ejaculates showed that both ejaculate analyses revealed results in the same order of magnitude and did not differ significantly from each other (*p* > 0.05). The correlation analysis revealed moderate to high positive correlation coefficients for the different sperm motility traits (Figure S2). Specifically, the measurements that were included as repeated observations in the GWAS showed a high correlation. Moreover, sperm with active mitochondria and sperm with intact acrosomes showed a phenotypic correlation of 89% in the dataset.

**Table 2:** Descriptive statistics of semen traits measured from the 4th and 5th ejaculates of 129 Piétrain boars.

Trait	Mean ± SEM		Min	Max
	4th Ejaculate	5th Ejaculate		
Fresh ejaculates:				
Ejaculate volume (mL)	202.5 ± 6.1	214.4 ± 6.6	17.0	408.0
Total sperm number	57.8 ± 1.7	59.4 ± 1.6	10.0	120.0
Sperm concentration (billion/mL)	0.306 ± 0.010	0.302 ± 0.011	0.055	0.874
Liquid-preserved AI doses:				
Motile sperm after storage at 17 °C (%)	83.9 ± 0.7	83.8 ± 0.6	15.5	97.8
Motile sperm after storage at 6 °C (%)	48.8 ± 1.4	52.1 ± 1.5	18.8	91.9
HRT ^1^: Motile sperm after 30 min 38 °C (%)	61.8 ± 2.2	63.7 ± 2.0	9.8	91.9
HRT: Motile sperm after 300 min 38 °C (%)	50.5 ± 2.1	53.0 ± 2.0	8.2	84.9
Morphologically normal sperm (%)	78.5 ± 1.4	77.3 ± 1.4	15.5	99.0
Droplet rate (%)	5.3 ± 0.6	6.1 ± 0.7	0.0	52.0
Sperm with intact plasma membrane and active mitochondria (%)	75.7 ± 0.7	75.2 ± 0.6	45.7	88.2
Sperm with intact plasma membrane and intact acrosome (%)	78.1 ± 0.6	77.1 ± 0.6	53.0	89.7

^1^ HRT = heat resistance test; data used for this table have partly been presented in Table 3 of [17].

**Table 3:** SNPs significantly associated with semen quality traits in a Piétrain population and their corresponding genomic regions and annotated genes.

Trait	SNP ID	Chr	Position (bp)	*p*-Value	Candidate Genes within 1 MB Window ^1^
Ejaculate volume	M1GA0007246	5	5216025	1.51 × 10^6^	*MCAT*, *MPPED1*, *PARVB*, *PARVG*, *PNPLA3*, *SAMM50*, *SCUBE1*, *SHISAL1*, *SULT4A1*, ***TSPO***, *TTLL12*
	ALGA0029934	5	5265052	1.14 × 10^5^	
	M1GA0007255	5	5290427	1.51 × 10^6^	
	ALGA0069136	13	26306761	4.43 × 10^5^	*ACKR2*, *ANO10*, *CCDC13*, *CCK*, *CYP8B1*, *HHATL*, *HIGD1A*, *KLHL40*, *LYZL4*, *NKTR*, *POMGNT2*, *SEC22C*, ***SNRK***, *SS18L2*, *TRAK1*, *VIPR1*, *ZBTB47*, *ZNF662*
Total sperm number	MARC0060820	1	231232620	2.34 × 10^5^	***CEP78***,***GNAQ***,***PSAT1***
	ALGA0008429	1	232226731	5.58 × 10^5^	***TLE4***
	ASGA0085355	3	679027	3.89 × 10^5^	*ADAP1*, *C7orf50*, *COX19*, *CYP2W1*,***DNAAF5***, *FAM20C*, ***FOXL3***, ***GPER1***, *INTS1*, *MAFK*, *MICALL2*, ***PDGFA***, ***PRKAR1B***, *PSMG3*, ***SUN1***, *TMEM184A*, *UNCX*, *ZFAND2A*
	ASGA0019946	4	64977285	5.69 × 10^5^	*LACTB2*, ***NCOA2***, *PRDM14*, *SLCO5A1*, *TRAM1*, *XKR9*
	ALGA0025512	4	65014643	5.69 × 10^5^	
	ALGA0049733	8	127722766	5.25 × 10^5^	***CCSER1***
Motile sperm after storage (17 °C)	ALGA0028043	4	109593443	3.18 × 10^5^	*AHCYL1*, *ALX3*, *CD53*, *CEPT1*, *CHI3L2*, *DENND2D*, *DRAM2*,***KCNA2***, ***KCNA3***, ***KCNA10***, ***KCNC4***, *LAMTOR5*, *LRIF1*, *PROK1*, *RBM15*, *SLC6A17*, *SLC16A4*, *STRIP1*
	ASGA0056281	13	14128868	5.67 × 10^5^	*EOMES*,*NEK10*, *SLC4A7*
Motile sperm after storage (6 °C)	MARC0001735	7	82578325	4.68 × 10^5^	***ARRDC4***, ***NR2F2***
	ALGA0042987	7	82605595	4.68 × 10^5^	
	ASGA0034705	7	82693734	4.68 × 10^5^	
	ASGA0034709	7	82746264	4.68 × 10^5^	
Motile sperm after HRT (38 °C)	MARC0024708	7	20074761	4.69 × 10^5^	*ARMH2*, *CARMIL1*, ***CMAH***, *GMNN*, *H2AC1*, *H2BC1*, *RIPOR2*, *SCGN*, *SLC17A1*, *SLC17A3*, *SLC17A4*
	ALGA0039273	7	20105440	4.69 × 10^5^	
	ASGA0091949	11	19893118	5.41 × 10^5^	*MED4*, *NUDT15*, ***SUCLA2***
Sperm with active mitochondria	MARC0086821	10	40750429	3.86 × 10^5^	*JCAD*, *MAP3K8*, ***MTPAP***, *SVIL*
	MARC0102352	10	41003878	3.13 × 10^5^	
Sperm with intact acrosome	MARC0086821	10	40750429	1.20 × 10^5^	*JCAD*, *MAP3K8*, ***MTPAP***, *SVIL*
	MARC0102352	10	41003878	7.53 × 10^7^	

^1^ Candidate genes within 1 Mb window: functional candidate genes (bold), positional candidate genes (underlined), upstream- and downstream-located genes (in italics only).

### 3.2. Genomic Regions and Candidate Genes Associated with Sperm Quality Traits

**Figure 1:**
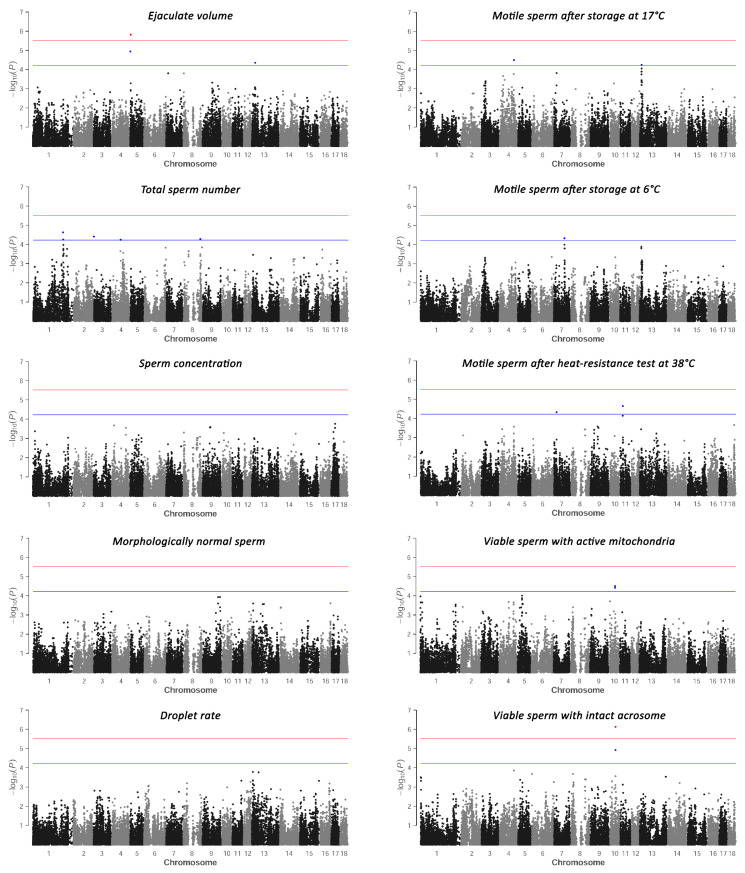
Manhattan plots of the semen quality traits indicating significant SNPs which passed the suggestive evidence threshold (blue) and genome-wide evidence threshold (red).

Significant associations of SNPs were observed for all the traits except the sperm concentration, sperm morphology, and droplet rate (Table 3, Figure 1 and Figure S3). The genomic regions are mainly indicated by single significantly associated SNPs but are usually supported by a peak pattern of other SNPs in the vicinity. Analysis of the ejaculate volume and viable sperm with intact acrosomes revealed significantly associated SNPs exceeding the genome-wide significance threshold. Table 3 shows the genomic positions of all the significantly associated SNPs with the respective traits and indicates the proposed positional and functional candidate genes in the corresponding genomic regions.

A GWAS of the ejaculate volume revealed three significantly associated SNPs pointing to a genomic region on chromosome 5. According to the annotation, one positional candidate gene (ENSSSCG00000060901) and one functional candidate gene (*TSPO*) were detected in the region around 5.25 Mb. Another SNP associated with the ejaculate volume was identified on chromosome 13 in the intronic region of *ACKR2*. For the total sperm number, four genomic regions on chromosomes 1, 3, 4, and 8 were identified. These regions comprise two positional candidate genes (*NCOA2* and *DNAAF5*) and seven functional candidates (*GNAQ*, *CEP78*, *PSAT1*, *NCOA2*, *PRDM14*, *FOXL3*, and *GPER1*).

The results on sperm motility after storage at 17 °C revealed two significantly associated SNPs located on chromosome 4 at 109.6 Mb and chromosome 13 at 14.1 Mb. Interestingly, the genomic region at the beginning of chromosome 13 was similarly visible in the analysis for the motility at 6 °C, although it was not statistically significant. The motility at 6 °C represents the cold resistance of sperm, which is an important trait for a possible reduction in antibiotics in semen extenders and was significantly associated with a cluster of four SNPs on chromosome 7 around 82.7 Mb in a long intergenic region between *ARRDC4* and *NR2F2*. The GWAS of the sperm motility after heat resistance testing at 38 °C revealed three significantly associated SNPs indicating two genomic regions on chromosome 11 at 19.9 Mb and chromosome 7 at 20.1 Mb. The former region harbors *SUCLA2* as a functional candidate gene, whereas the SNPs indicate the latter region map in the *CARMIL1* gene.

In the analysis of sperm with active mitochondria and sperm with intact acrosomes, the same two SNPs showed suggestive evidence of an association and are located around 41 Mb on chromosome 10.

## 4. Discussion

A GWAS was conducted to identify genomic regions and candidate genes associated with the boar parameters and phenotypes derived from informative but complex sperm motility tests in a population of young Piétrain boars. Within the genomic regions on chromosome 5, the translocator protein-encoding gene *TSPO* was identified as a functional candidate gene for ejaculate volume. TSPO is a cholesterol-binding protein that shows high expression in steroidogenic tissues and mediates the transport of cholesterol into the mitochondria. TSPO has been suggested to play a critical role in germ cell development, spermatogonial stem cell formation, spermatogonial mitosis, and spermatogenesis [23,24]. In a genome-wide study in cattle, *TSPO* was proposed as a candidate gene for spermatogenesis [25]. The positional candidate gene in the region at 5.2 Mb on chromosome 5, ENSSSCG000060901, has not yet been annotated in pigs but shows sequence similarities to *EFCAB6* in cattle, which is thought to play a role in transcription activity regulation of the androgen receptor [26]. For the genomic region significantly associated with ejaculate volume on chromosome 13, *SNRK* and *ANO10* appear to have relevant functions. The *SNRK* gene is a SNF1-related kinase with a considerable expression level in the testis. It is activated by liver kinase B1 (LKB1) and in this context is suggested to play a role in sperm release from the germinal epithelium [27,28]. The *ANO10* gene (*TMEM16K*) is a member of the TMEM16 family of ion channels, of which the members TMEM16A and TMEM16B have been linked to sperm motility and fertilization [29].

The GWAS of the total sperm number revealed several associated genomic regions. In the regions on chromosome 1, *GNAQ*, *CEP78*, *PSAT1*, and *TLE4* may be the most relevant candidates. *GNAQ* has been reported to be involved in ovine gonadal development and sperm maturation and is significantly associated with seasonal reproduction and litter size in Kazakh and Chinese Merino sheep [30,31]. *CEP78* is a centriole wall protein involved in the regulation of centrosome duplication and is generally expressed in cilia-containing organisms, suggesting a function in cilia biogenesis [32,33]. It is associated with male infertility in humans, where a pathogenic splice mutation in *CEP78* was identified in patients with severely reduced sperm counts and motility, as well as multiple sperm morphological abnormalities [34,35]. The gene *PSAT1* encodes phosphoserine aminotransferase 1. In humans, a *PSAT1* SNP has been described, which was significantly associated with male infertility traits and might explain to some extent the genetic contribution to severe oligospermia [36]. The *TLE4* gene encodes a transcriptional corepressor involved in Wnt signaling. It shows testis-specific splicing variants [37] and has been associated with the genetic variance in total sperm motility and progressive motility in Italian Holstein Bulls [38]. The region at the beginning of chromosome 3 harbors a number of genes that may be related to total sperm number through their described roles in spermatogenesis (*FOXL3*, *GPER1*, *PDGFA*, *PRKAR1B*, *SUN1*) [22,39,40] and sperm motility (*DNAAF5*, *GPER1*) [22,40]. From a functional perspective, the G protein-coupled estrogen receptor 1, encoded by *GPER1*, appears to be the most relevant candidate in this genomic region. *GPER* expression has been found in the spermatozoa of pigs and humans [41]. The serum levels of GPER in male patients correlated positively with total sperm count, sperm concentration, motility, and morphology, indicating a promising role in the study of male infertility [40]. On chromosome 4, the nuclear receptor coactivator (*NCOA2*) gene is proposed as a functional candidate gene affecting the sperm number. In previous association studies in zebus and European cattle, *NCOA2* has been linked to the onset of puberty and some female reproductive traits [42,43]. Moreover, low expression of the *NCOA2* gene was detected in subfertile Brown Norway rats [44]. The genomic region detected on chromosome 8 points to *CCSER1*, encoding Coiled Coil Serine-Rich Protein 1. This gene region represents a selection signature discriminating between different sheep breeds and is associated with sperm count in Assaf sheep and sperm quality in Holstein-Friesian bulls [45,46,47].

For the sperm motility at 17 °C, the significantly associated SNP on chromosome 4 was located in a region with several members of subfamily A of voltage-gated potassium channels (*KCN*). Voltage-gated potassium channels play a role in regulating the osmolality and membrane potential in spermatocytes, although no specific functional relationships with subfamily A members (i.e., KCNA2, KCNA10, KCNA3) have yet been established [29]. However, a member of the subfamily Q potassium channels was shown to be expressed in human sperm and to regulate their motility [48]. The second significantly associated SNP for this trait points to NEK10 as a positional candidate on chromosome 13. NEK10 is related to cilia assembly but has not been associated with sperm so far [49].

The association analysis with sperm motility at 6 °C revealed the two genes *ARRDC4* and *NR2F2*. The protein encoded by *ARRDC4* affects the biogenesis of extracellular vesicles and is therefore associated with sperm maturation [50]. An Arrdc4-/- mice model showed impaired overall motility and sperm function, culminating in a male subfertility phenotype. *NR2F2* plays a crucial role in steroidogenesis, the differentiation of Leydig cells, and male fertility, in particular through the activation of the steroidogenic acute regulatory protein (STAR) [51,52].

The GWAS of the sperm motility after heat resistance testing at 38 °C proposed two functional candidate genes which might affect the sperm surface. On chromosome 11, *SUCLA2* encodes a mitochondrial enzyme involved in the tricarboxylic acid cycle, which is putatively related to morphological and metabolic changes in mammalian sperm capacitation [53]. Moreover, the SUCLA2 protein expression in the sperm differed under cryopreservation conditions compared to that in fresh sperm, suggesting a role in capacitation-like changes in sperm under storage conditions [54]. The cytidine monophospho-N-acetylneuraminic acid hydroxylase (CMAH, encoded by ENSSSCG00000001099), located on chromosome 11, is involved in the biosynthesis of the sialic acid N-Glycolylneuraminic acid (Neu5Gc). In fact, the spermatozoa are coated with sialic acids, which affect the surface of the sperm and are thought to interfere with sperm survival and fertilization [55].

The GWAS for mitochondrial activity and acrosomal integrity highlighted the same region on chromosome 10. This might be due to the high phenotypic correlation of both traits [56]. This genomic region harbors a number of annotated genes, including *MAP3K8*, *MTPAP*, *JCAD*, and *SVIL*. Of these, *MTPAP*, which is involved in the processing of mitochondrial RNA, was described to differ in gene expression between summer and winter ejaculates from boars [57].

This is the first study on the genetics of complex sperm motility parameters derived from semen stress tests, which limits comparison of the results with other studies. These parameters go beyond routine sperm analysis by assessing the functionality and resilience of the sperm under stress conditions, such as those encountered during the implementation of AI, which is crucial for predicting fertility. The use of these advanced parameters allows for a more comprehensive assessment of sperm quality and its impact on fertility, with genetic analysis enhancing our understanding of the traits related to male reproductive capacity. There are only a few holistic studies on the genetics of sperm parameters in Piétrain boars. Candidate gene approaches revealed significant associations with respective traits in Piétrain boars, including Actin Gamma 2 (*ACTG2*), gonadotropin-releasing hormone receptor (*GNRHR*), and inhibin beta A (*INHBA*) [58,59]. Recently, an integrative approach was used to identify genetic markers and candidate genes in a population of 300 Piétrain boars [60]. However, overlaps in the QTL regions with the current study were only found for the region on chromosome 13. In general, the difficulty in genetic analysis for such elaborate but informative parameters is achieving large sample sizes. Given the small sample size of the current dataset and despite the effort required to perform semen stress testing, replication studies in independent Piétrain populations would be critical to validate the findings and ensure the robustness of the GWAS results. By strategically handling repeated observations of consecutive boar ejaculates and using a GWAS approach suitable for this purpose, the reliability of the study was maximized at both the phenotype and analysis levels.

## 5. Conclusions

The results of this study provide insights into the genetics of sperm quality traits in young Piétrain boars. The GWAS revealed several genomic regions represented by 21 significantly associated SNPs. Candidate genes were identified with functions in spermatogenesis and sperm motility, whereas other candidates lack functional annotations for establishing explicit links with the analyzed phenotypes. Some of the genes presented, such as *CCSER1*, *GNAQ*, *NCOA2*, *SNRK*, and *TSPO*, have been previously associated with fertility traits in livestock species or related to infertility in human patients (*CEP78* and *GPER1*). Genes with functions related to the sperm coating and morphology (e.g., *SUCLA2*, *CMAH*) might contribute to differences in the sperm motility and environmental resistance of sperm. Consequently, the analyses revealed genetic regions and genes that deserve further research and consideration when distinguishing the reproductive performance of boars.

## Figures and Tables

**Table 1:** Traits and recording time points of the semen quality characteristics (detailed information on trait analysis in [17]) for the 4th and 5th ejaculates of 129 boars.

Trait	Observation Time Point
Fresh ejaculates:	
Ejaculate volume (mL)	Directly after semen collection
Total sperm number (bn)	Directly after semen collection
Sperm concentration (bn/mL)	Directly after semen collection
Liquid-preserved AI doses:	
Motile sperm after storage at 17 °C (%)	Storage days 1, 2, 3
Motile sperm after storage at 6 °C (%)	Storage day 3
Motile sperm after HRT ^1^ (%)	Storage day 3 (17 °C); 30 and 300 min at 38 °C
Morphologically normal sperm (%)	Storage day 2 (17 °C)
Droplet rate (%)	Storage day 2 (17 °C)
Viable sperm with active mitochondria (%)	Storage day 2 (17 °C)
Viable sperm with intact acrosome (%)	Storage day 2 (17 °C)

^1^ HRT = heat resistance test.

## Data Availability

The raw data supporting the conclusions of this article will be made available by the corresponding authors on request.

## Supplementary Materials

The following supporting information can be downloaded at https://www.mdpi.com/article/10.3390/genes15030382/s1. Figure S1: Density plot representing the distribution of phenotypic observations in the Piétrain boar population. The dashed lines indicate the cut points at 75% and 40% for the dichotomization of sperm motility at 17 °C and at 38 °C, respectively; Figure S2: Pearson’s correlation matrix between measurements of semen quality traits in a population of Piétrain boars; Figure S3: Quantile–quantile plots for the GWAS of semen quality traits in a population of Piétrain boars.
