# Supplementary figures and images for: Genome-Wide Association Analysis of Semen Characteristics in Piétrain Boars
Source: Genes (Basel). 2024 Mar 20;15(3):382. doi: 10.3390/genes15030382 (PMC10969825; doi:10.3390/genes15030382)

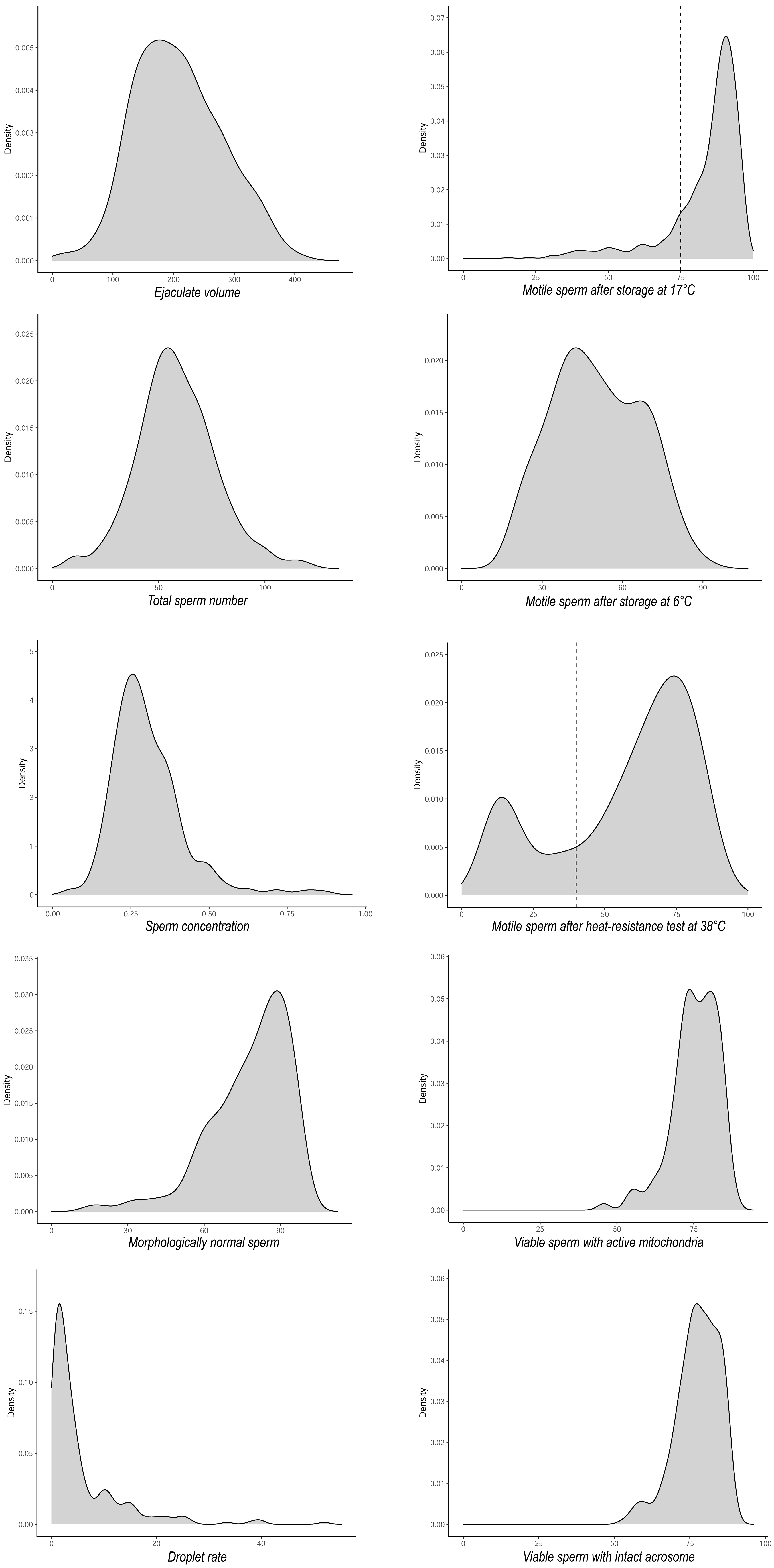

Supplement: Supplementary file 1 [file genes-15-00382-s001.zip › Figure_S1_Density_Plots.tif]

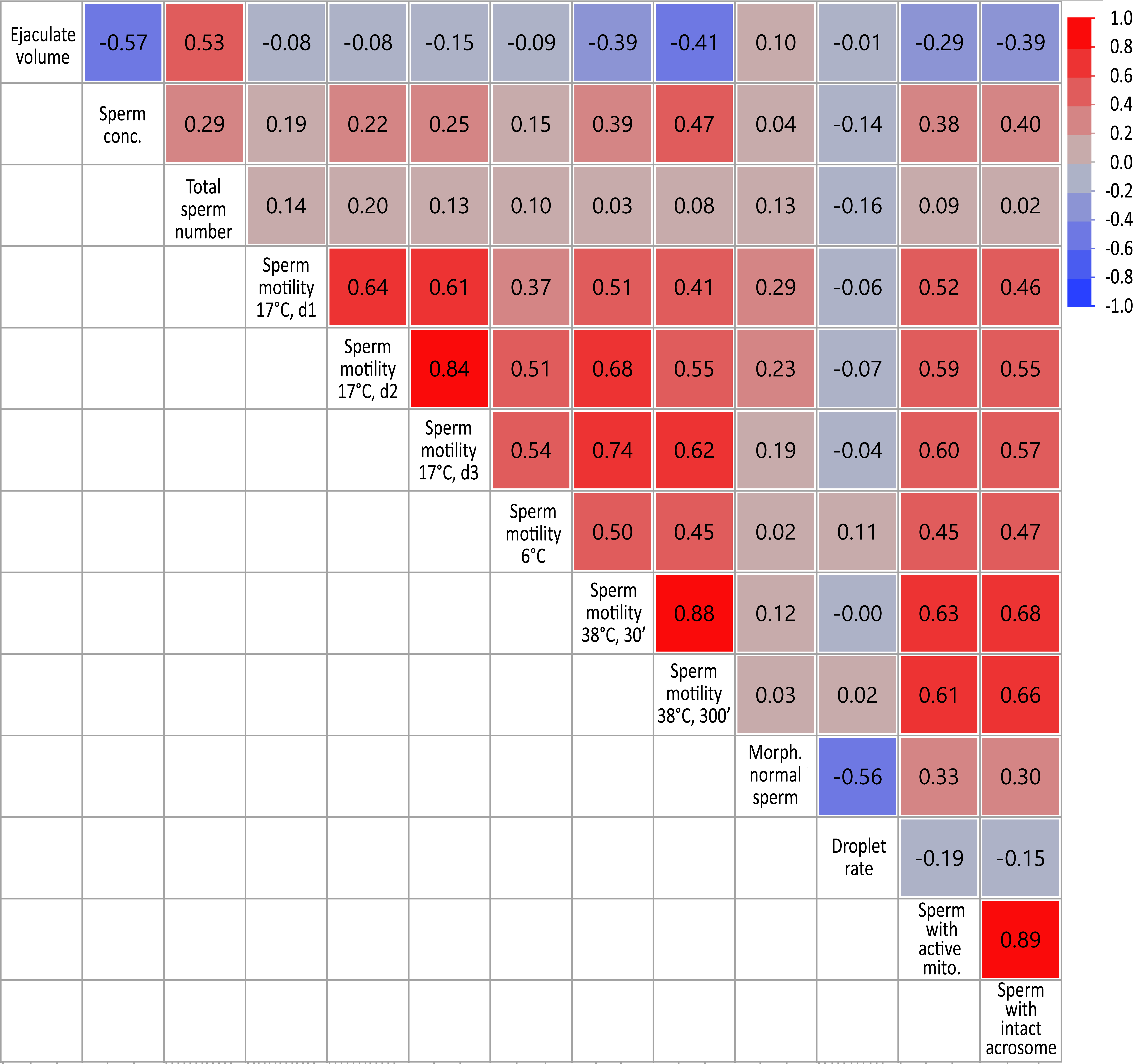

Supplement: Supplementary file 1 [file genes-15-00382-s001.zip › Figure_S2_Correlation.tif]

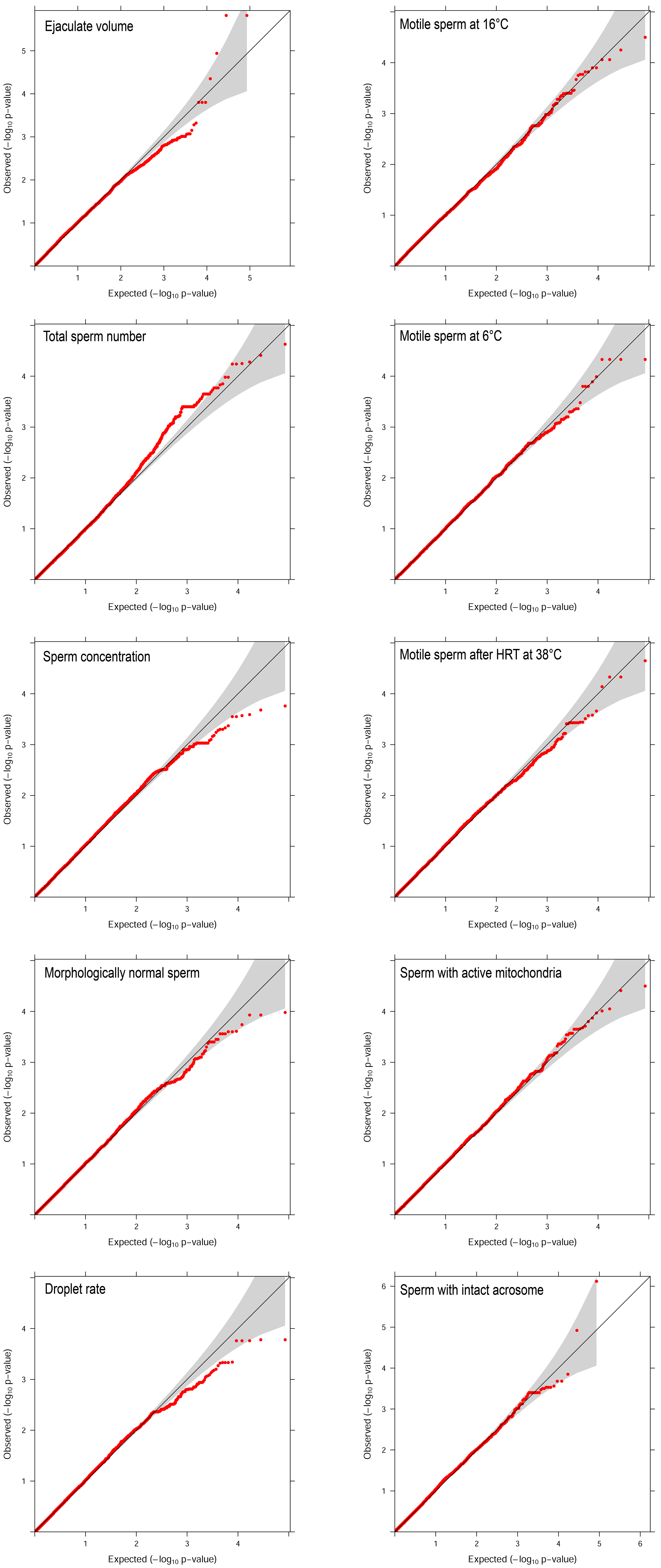

Supplement: Supplementary file 1 [file genes-15-00382-s001.zip › Figure_S3_QQ_Plots.tif]
